# Attention Guides the Motor-Timing Strategies in Finger-Tapping Tasks When Moving Fast and Slow

**DOI:** 10.3389/fpsyg.2020.574396

**Published:** 2021-01-25

**Authors:** Ségolène M. R. Guérin, Juliette Boitout, Yvonne N. Delevoye-Turrell

**Affiliations:** University of Lille, CNRS, UMR 9193 - SCALab - Sciences Cognitives et Sciences Affectives, Lille, France

**Keywords:** spontaneous motor tempo, sensorimotor synchronization, dual task, finger tapping, autocorrelations, motor timing, timing strategies

## Abstract

Human beings adapt the spontaneous pace of their actions to interact with the environment. Yet, the nature of the mechanism enabling such adaptive behavior remains poorly understood. The aim of the present contribution was to examine the role of attention in motor timing using (a) time series analysis, and (b) a dual task paradigm. In a series of two studies, a finger-tapping task was used in sensorimotor synchronization with various tempi (from 300 to 1,100 ms) and motor complexity (one target vs. six targets). Time series analyzes indicated that two different timing strategies were used depending on the speed constraints. At slow tempi, tapping sequences were characterized by strong negative autocorrelations, suggesting the implication of cognitive predictive timing. When moving at fast and close-to-spontaneous tempi, tapping sequences were characterized by less negative autocorrelations, suggesting that timing properties emerged from body movement dynamics. The analysis of the dual-task reaction times confirmed that both the temporal and spatial constraints impacted the attentional resources allocated to the finger-tapping tasks. Overall, our work suggests that moving fast and slow involve distinct timing strategies that are characterized by contrasting attentional demands.

A captivating property of human behavior is that most of our everyday life actions share a similar temporality. In the past 50 years, psychological sciences have confirmed the existence of a preferred tempo, referred as the *spontaneous motor tempo* (SMT), which is the most natural and easiest pace to move (Fraisse et al., 1954; Fraisse, 1974). Among the human species, the SMT is found to average ~2 Hz in adult populations (Moelants, 2002; McAuley et al., 2006). This particular motor signature is slightly faster in children and slower in elderly individuals, but remains strikingly close to two movements per second, even across tasks of different levels of complexity (e.g., finger tapping, foot stomping, hand clapping). Delevoye-Turrell et al. (2014) reported that repetitive cyclic movements (e.g., finger-tapping and cycling tasks) were accomplished with greater accuracy and better stability when performed at the SMT compared to execution at faster or slower tempi. Interestingly, studies on locomotion (e.g., during walking or running) indicated that the SMT is also associated with a minimum metabolic energy consumption (Sparrow and Newell, 1994; Holt et al., 1995). Indeed, moving faster or slower than the SMT were activities associated with greater energy expenditure. This phenomenon may be due to the fact that the ability to modulate the pace of spontaneous motor behaviors requires control.

Experimental evidence has revealed a clear ability of human beings to synchronize the pacing of their actions with external events (Bryant and Barrett, 2007; Kirschner and Tomasello, 2009). With experience, children learn to adapt their SMT to match the pace of surrounding entities, such as their parents' behaviors (Brazelton et al., 1975). Nonetheless, this ability is not innate. Bobin-Bègue and Provasi (2008) reported that very young children (1½- and 2½-years-old) were capable neither to accelerate nor to decelerate the pace of their action, confirming that adjustments in motor timing required some sort of cognitive control that was not yet available to them. Interestingly, 3½-years-old were able to accelerate but not slow down the pacing of their movements, that is they were able to tap in rhythm with fast-paced auditory metronomes but not with slow ones. This pattern of results indicated that the capacity to slow down the tempo of voluntary movements appears later in life than the capacity to accelerate. This developmental asymmetry described in children may be due to the later maturation of the frontal executive functions, which would be needed to decelerate self-initiated actions according to externally-imposed metronomes (Provasi and Bobin-Bègue, 2003). More specifically, inhibitory capacities would be involved to stop the urge to move as fast as the SMT. This phenomenon is experienced for example during downhill walking, for which the pace of walking can increase through the simple dynamic shift of body weight.

In cognitive psychology, early studies claimed that the pacing of motor behaviors was underpinned by a central temporal mechanism, seen as a general skill (Keele et al., 1985; Franz et al., 1992). With a resonance of such thinking, the model of the *internal clock* conceptualized the processing of time using cognitive entities: a pacemaker, a counter, a store, and a comparator (Treisman, 1963). This pacemaker-accumulator model would be composed of three distinct stages in which temporal informations are extracted, encoded, and processed. In addition, this model includes a motor component to account for peripheral variance (Wing and Kristofferson, 1973b). Accordingly, time production can be modeled following Equation (1):

(1) $$\begin{array}{l} I_{i}=C_{i}+M_{i+1}-M_{i} \end{array}$$

where *i* is a time interval, *I* is the series of time intervals, *C* is a central source of variance related to the generation of time intervals by the internal clock, and *M* is a peripheral source of variance that reflects motor delay.

Motor timing would depend on four cognitive components that would adjust the production of voluntary-motor actions on the basis of an explicit representation of time interval, a process that is cognitive and thus, requires attentional resources. However, the results reported by Provasi and Bobin-Bègue (2003) strongly suggest the existence of an asymmetry in the development of temporal control processes, with an earlier maturation of functions for motor acceleration than for motor deceleration. This asymmetry in timing abilities for slow and fast actions in children could be due to the involvement of two separate timing strategies.

Over the past two decades, the existence of two temporal control processes has been advocated (Robertson et al., 1999; Zelaznik et al., 2000). Zelaznik et al. (2002) distinguished the explicit from the implicit strategies, later renamed, respectively *predictive* and *emergent* timing (Ivry et al., 2002; Spencer and Ivry, 2005). While predictive timing would rely on the internal clock model (Treisman, 1963), emergent timing would depend on the implicit emergence of temporal regularities, from the kinetic parameters inherent to body dynamics (e.g., mass, length, velocity; Zelaznik et al., 2002). From the dynamical system approach, interval timing would be the result of the interaction between the individual, her/his environment and the physical constraints of the behavioral task. In order to model emergent timing, Delignières et al. (2004) proposed Equation (2):

(2) $$\begin{array}{l} I_{i}=D_{i}+\xi_{i} \end{array}$$

where *i* is a time interval, *I* is the series of time intervals, *D* is the self-sustained oscillatory frequency, and ξ is a Gaussian white noise accounting for the variability inherent to biological systems.

A striking difference between the two timing strategies is their degree of capacity to correct timing errors. Indeed, within the internal model theoretical frame, cognitive control is required for the detection and implementation of error corrections. Hence, only the predictive timing mode should be in the capacity to correct timing errors during ongoing motor activities. It is the case that autocorrelation (AC) analyses have been used to confirm the complementary role of the two timing strategies as a function of temporal and/or spatial constraints set upon the performance of motor activities. In bimanual oscillatory movements (as in circle drawing), a predominancy of emergent timing was reported (Wing and Kristofferson, 1973a; Vorberg and Wing, 1996). As emergent timing does not support a correction process, the series of time intervals (i.e., inter-response intervals, IRIs) are characterized by positive or close-to-zero ACs (Robertson et al., 1999; Studenka and Zelaznik, 2008). Thus, a too short *n* interval can be followed by an even shorter *n* + 1 interval. On the other hand, tasks affording predictive timing (as in tapping tasks) are characterized by the presence of error-correction mechanisms: A too short *n* interval is followed by a longer *n* + 1 interval, and vice versa. The IRI series are in this case characterized by negative ACs. Therefore, temporal strategies can be distinguished at a behavioral level according to the shape of their error distributions, which would reflect the nature of the underlying timing strategies.

Emergent and predictive timing are generally described as mutually exclusive strategies, depending on the features of the performed movements (Robertson et al., 1999): Predictive timing is peculiar to discrete movements (i.e., with recognizable beginning and end), and emergent timing to continuous movements (i.e., with no recognizable beginning and end; Schmidt et al., 1988). Yet, it has been suggested that the task is not a key-point in the distinction between the two timing strategies, since some behavioral tasks can alternatively appeal to one timing mode or the other (Huys et al., 2008; Madison and Delignieres, 2009). An example entails the air-tapping task, which is voluntary tapping movements without contact with a surface. When participants were required to perform this task at their SMT, Delignieres and Torre (2011) reported that the temporal control mode was emergent (i.e., positive ACs), predictive (i.e., negative ACs) or hybrid (i.e., close-to-zero ACs) according to the timing strategy adopted by each individual. Accordingly, the use of one or other timing strategies is dependent not on the *nature* of the task, but on the *way* participants perform the task—whether they use or not an explicit representation of the temporal intervals.

The time pressure set upon the execution of a voluntary movement may be a factor that orients the timing strategy used. During an air-tapping task in synchronization with a metronome, Huys et al. (2008) reported that movements were less discrete when an external metronome constrained participants to increase motor tempo. Similar results were found using a *spatial finger-tapping task*, which is an hybrid task that combines the requirements of the finger-tapping and circle-drawing tasks (Dione and Delevoye-Turrell, 2015). When required to tap successively on six targets arranged in a circle following the pace given by a metronome, participants were found to use emergent timing for fast tempi (i.e., faster than the SMT), and predictive timing for slow tempi (i.e., slower than the SMT). Thus, the alternation of the two timing modes depended on the temporal constraints inherent to the task. Nevertheless, ACs were computed over the entire trial, which is more prone to bias than moving-average time series analysis (Delignieres and Torre, 2011). In addition, ACs analysis alone is not sufficient to shed light on the cognitive nature of the processes that underlie the temporal strategies.

The aim of the present set of studies was to examine the role of the cognitive-control system in the alternation of emergent and predictive timing using (a) time series analysis, and (b) a dual-task paradigm to index the attentional demands of the task. The classic finger-tapping task (simple 1-target pattern) and the spatial finger-tapping task (complex 6-target pattern) were used to assess the effects of both time constraints and motor complexity on the alternating use of predictive and emergent timing strategies. The two finger-tapping tasks were administered via a computer touchscreen according to externally-paced tempi ranging from 300 to 1,100 ms. In Study 1, time series analysis (detrend windowed autocorrelations, DWA; Lemoine and Delignières, 2009) was used to confirm the alternating involvement of predictive and emergent timing as a function of motor tempo. We hypothesized that slow tempi would promote predictive timing (i.e, negative lag-1 ACs), whereas fast tempi would endorse emergent timing (i.e., positive or close-to-zero lag-1 ACs; *H*1). The complexity of the motor task being undertaken would not significantly impact the use of one or other of the timing strategies (*H*2). In Study 2, a dual-task paradigm was designed to reveal the amount of attentional resources needed to perform the finger-tapping task under contrasting time and task complexity constraints. We hypothesized that action production at slower tempi would lead to longer reaction times when compared to task execution at faster tempi (*H*3). Furthermore, finger tapping would be associated to shorter reaction times when pointing to the 1-target than the 6-target visual pattern, as the later requires the control of hand movements through space and time (*H*4).

## 1. Study 1

Autocorrelation (AC), sometimes known as *serial correlation*, is the correlation of a time series with a delayed copy of itself as a function of time. That is, it measures the similarity between observations as a function of the time lag between them. The AC function can be used to detect non-randomness in the data, and to identify cyclical patterns, if any. In the present case, the aim was to reveal cyclic patterns of time intervals produced in synchrony with an external pacing metronome. AC is basically a Pearson correlation coefficient, but instead of calculating it between two different variables, it is calculated between two values of the same variable at two distinct moments in time, *X*_*t*_ and *X*_*t*+*k*_. The resulting values are usually plotted for different lags *k* in a so called correlogram.

The use of AC analysis in timing research was originally proposed by Wing and Kristofferson (1973a) to measure the variance of predictive model components. Thereafter, Lemoine and Delignières (2009) adjusted the mathematical approach to develop the DWA method that provides the means to reveal the use of emergent and predictive timing in short time series (i.e., 128 data points), with less bias and variability than other similar techniques (e.g., spectral analysis; Delignieres et al., 2006). Using the DWA method, Delignieres and Torre (2011) were able to highlight positive windowed lag-1 ACs in continuous movements, and negative windowed lag-1 ACs in discrete movements.

In the present study, the DWA method was used to confirm that two different timing strategies are implemented as a function of the constrained speed of motor execution. Indeed, if two different motor timing strategies are used to produce pointing actions at fast and slow pace, the modeling of the redundant cyclic patterns through DWA should provide distinct patterns of time series.

### 1.1. Materials and Methods

#### 1.1.1. Participants

The sample size required for the present study was calculated using G*Power (3.1.9.2). The theoretical sample size was computed for a repeated-measured analysis of variance (RM ANOVA), with the lag-1 ACs results of Dione and Delevoye-Turrell (2015) as group parameters. The power analysis indicated that a minimum of 20 participants were required (*f* = 0.56; α = 0.05; 1-β = 0.80). An additional five participants were recruited in case of deletions due to outliers.

Twenty five right-handed participants between 18 and 35 years (*M* = 21.6, *SD* = 1.4) participated voluntarily in the study. Each of them received an information sheet, and completed a written informed consent. Participants reported having normal or corrected-to-normal vision and no deficiencies in terms of motor control.

The small telescopes approach was used to determine the smallest effect size of interest (SESOI; i.e., the difference that is considered too small to be meaningful; Simonsohn, 2015). Accordingly, the SESOI was set to the effect size that an earlier study would have had 33% power to detect (Lakens et al., 2018). As previously, the lag-1 ACs results of Dione and Delevoye-Turrell (2015) were used as group parameters. The sensitivity analysis indicated that an effect size of at least *f* = 0.09 (i.e., $\eta_{p}^{2}$ = 0.01) was required to be meaningful.

#### 1.1.2. Tasks Description and Materials

Participants were administered two finger-tapping tasks on a touchscreen using the right index finger, with a closed fist. The touchscreen (1915L Elo Touch 19″; Elo Touch Solutions Inc.; Milpitas, CA) was placed on a table in front of the participant, with the screen oriented at 45°. The participant was seated on a stool, which was suitably adjusted to her/his height to minimize muscular fatigue and optimize comfort.

**Figure 1:**
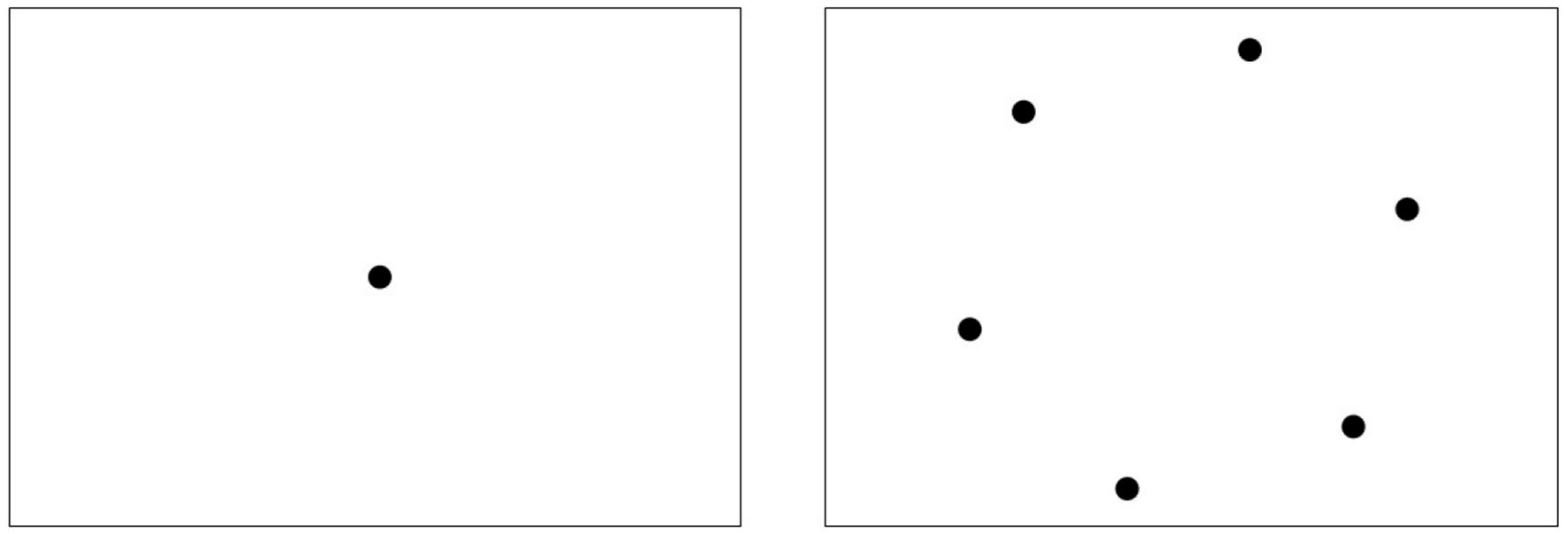
Experimental materials. Illustrations of the visual stimuli for the 1-target (left), and the 6-target conditions (right).

In the 1-target condition, the participant was required to tap on a single target (10-mm-diameter black dot) displayed in the center of the screen (Figure 1, left panel). In the 6-target condition, six targets (10-mm-diameter black dot) arranged in a circle pattern (100-mm radius) were displayed on the screen (Figure 1, right panel). The participant was instructed to tap each target one after the other starting from the top-right target, and moving counterclockwise.

In both tasks, the participant was asked to synchronize her/his finger taps with the auditory cues given by a metronome. The beeps of the metronome (duration = 80 ms; sound frequency = 294 Hz) were played through Creative SBS 250 desk speakers that were placed on both sides of the screen. The beeps indicated inter-stimuli intervals (ISIs) of either 300, 450, 600, 800, or 1,000 ms. These fast and slow metronome paces enabled the participant to depart from her/his SMT, but to remain within the possible sensorimotor synchronization zone (between 180 and 1,800 ms; Keele et al., 1985). The auditory stimuli were generated using MATLAB 7.11.0 R2010 software (Mathworks Inc.; Natick, Massachusetts, MA).

#### 1.1.3. Procedure

A within-subjects design was applied wherein each finger-tapping task was performed in a fully counterbalanced order. For each of the two tasks, ISIs were presented with increasing time intervals on each task for half of the participants (i.e., from 300 to 1,000 ms), and with decreasing time intervals for the other half (i.e., from 1,000 to 300 ms). A trial consisted in 180 beeps. Overall, participants performed a total of 10 trials. The total duration of the experimental test period was ~45 min. Participants were systematically debriefed at the end of the session.

#### 1.1.4. Data Acquisition and Processing

To promote an “efficient, progressive, and ultimately self-correcting scientific ecosystem that generates credible findings” (Hardwicke et al., 2018, p. 2), all collected data are available as Supplementary Material.

##### 1.1.4.1. Inter-response Intervals

IRIs were measured as the time interval between the onset of successive taps. Before calculating accuracy indicators and windowed lag-1 ACs, the series of taps were checked to detect and remove the IRIs greater than twice the ISI of a given trial. Overall, 0.16% of the data were removed from the analysis. These trials were referred to as temporal omissions, and were not included in further analyses.

##### 1.1.4.2. Accuracy Indicators

To confirm that participants performed the finger-tapping tasks accurately, timing and spatial errors were computed. Relative asynchrony (ms) was calculated as the absolute time difference between the tap and the beep, divided by the ISI. Thus, the relative absolute asynchrony within a trial was used as an indicator of synchronization accuracy that takes into account the scalar property of timing (i.e., larger time intervals generate more errors; see Rakitin et al., 1998).

Spatial error (pixels) was computed as the difference between the center of the visual target and the location of the participant's fingertip. The mean pointing error within a trial was used as an indicator of spatial accuracy.

##### 1.1.4.3. Detrend Windowed Autocorrelations

The DWA procedure was used to reveal the evolution of lag-1 ACs within a trial (Lemoine and Delignières, 2009). This procedure provided the means to reduce the frequent bias and high variability observed within lag-1 ACs (Delignieres et al., 2006). Windowed lag-1 ACs were computed for each individual trial as follows: A window corresponding to the first 30 IRIs of a trial was selected, and the linear trend of this window was removed. Then, the lag-1 AC was calculated. This procedure was repeated after shifting the window by one IRI event. This method was applied until the moving window had scanned the entire time series. A total of 150 windowed lag-1 ACs were computed per trial. Finally, the mean windowed lag-1 AC was computed, and used as an indicator of the dominant timing mode used for a given trial.

#### 1.1.5. Statistical Analysis

To monitor motor performance in the two visuomotor tasks, absolute asynchronies and spatial errors were submitted to a twoway, 2 (Task [1 target, 6 targets]) × 5 (ISI [300, 450, 600, 800, 1,000 ms]), RM ANOVA. To examine *H*_1_ and *H*_2_, the mean windowed lag-1 ACs were also submitted to a twoway RM ANOVA. Normality was checked using the Shapiro–Wilk test. Where Mauchly's tests indicated violations of the sphericity assumption, Greenhouse–Geisser adjustments were applied. Tukey *post-hoc* tests were used as necessary. Statistica (v.13.1) was used for the statistical analyses, and the alpha level was set at *p* < 0.05.

### 1.2. Results

#### 1.2.1. Descriptive Results

Mean absolute asynchronies and spatial errors are presented in Table 1. Overall, participants were able to perform accurately the two visuomotor tasks following both time and space constraints.

**Table 1:** Synchronization and spatial indicators for Study 1.

**Indicator**	**1-target pattern**					**6-target pattern**				
	**300 ms**	**450 ms**	**600 ms**	**800 ms**	**1,000 ms**	**300 ms**	**450 ms**	**600 ms**	**800 ms**	**1,000 ms**
Relative asynchrony	110	113	115	116	95	149	133	157	153	120
Spatial error	7.8	7.7	8.4	8.4	8.8	16.3	10.5	9.1	7.8	7.8

*Relative asynchrony (ms) and spatial error (pixel) for each inter-stimuli interval and each task*.

##### 1.2.1.1. Synchronization Accuracy

The main effect of the task was significant, *F*_(1, 24)_ = 11.10, *p* = 0.003, $\eta_{p}^{2}$ = 0.32, with a smaller relative asynchrony in the 1-target (*M* = 110, *SD* = 65) than in the 6-target condition (*M* = 142, *SD* = 83). Neither the main effect of ISI nor the ISI × Task interaction were significant. Overall, these results revealed that the participants made more timing errors when the motor task was complex.

##### 1.2.1.2. Spatial Accuracy

The RM ANOVA revealed a significant main effect of the ISI, *F*_(4, 96)_ = 55.26, *p* < 0.001, $\eta_{p}^{2}$ = 0.70, with smaller spatial errors in the 600, 800, and 1,000 ms ISI conditions (*M* = 8.4, *SD* = 2.6) than in the 450 ms (*M* = 9.1, *SD* = 2.1) and in the 300 ms ISI conditions (*M* = 12.0, *SD* = 2.9). The main effect of the task was also significant, *F*_(1, 24)_ = 32.58, *p* < 0.001, $\eta_{p}^{2}$ = 0.58, with smaller spatial errors in the 1-target (*M* = 8.2, *SD* = 3.0) than in the 6-target condition (*M* = 10.3, *SD* = 2.1). The ISI × Task interaction was significant, *F*_(4, 96)_ = 72.39, *p* < 0.001, $\eta_{p}^{2}$ = 0.75. This interaction reflected that spatial errors were smaller in the 1-target than in the 6-target condition, but only in the 450 ms (*p* < 0.001) and in the 300 ms ISI conditions (*p* < 0.001); however, pointing errors were greater in the 1-target than in the 6-target condition in the 1,000 ms ISI (*p* = 0.030). Overall, these results indicated that the participants made more spatial errors when the motor task was complex, particularly when the speed of execution was fast.

#### 1.2.2. Mean Windowed Lag-1 ACs

**Figure 2:**
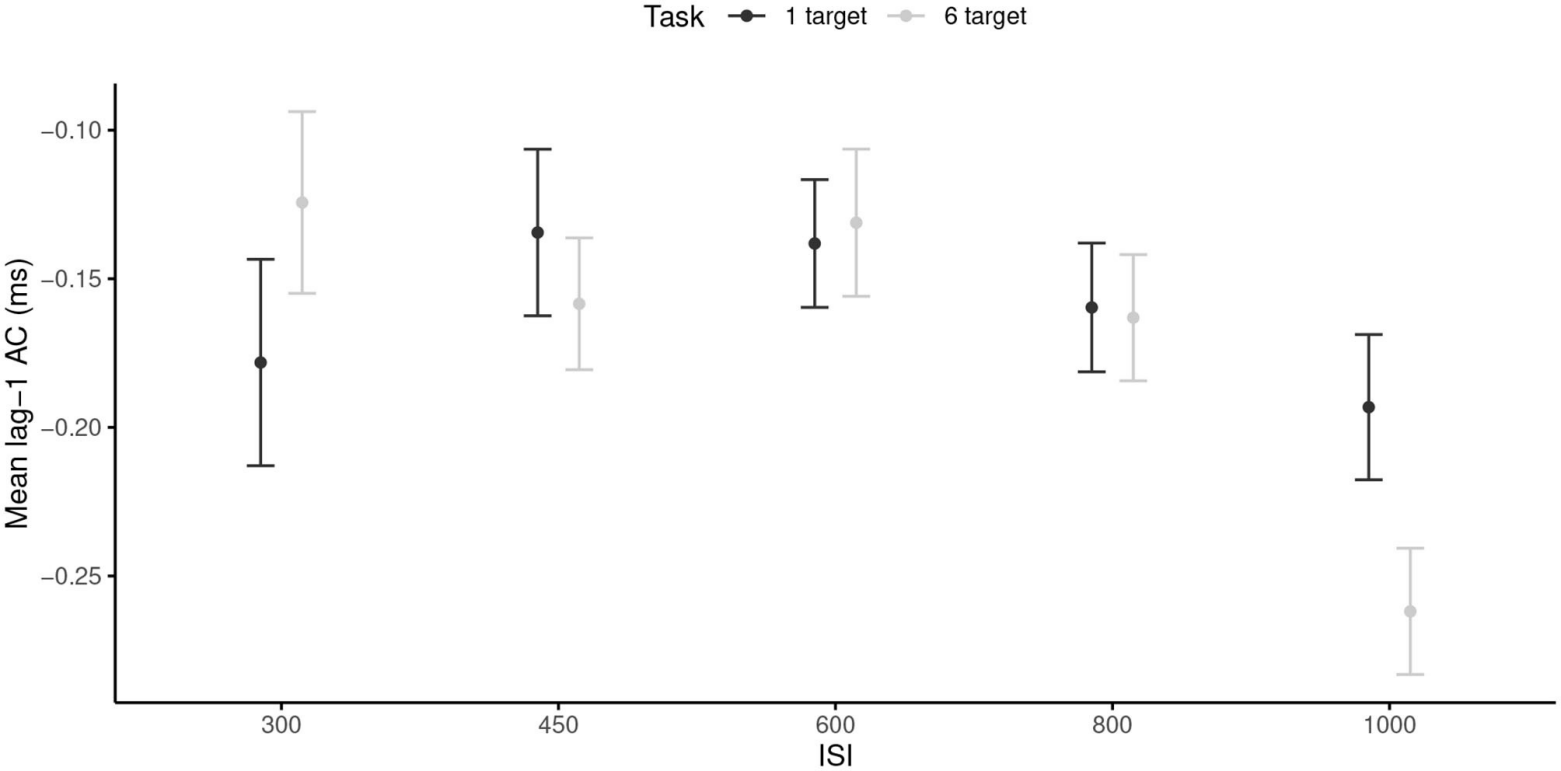
Mean lag-1 ACs for each inter-stimuli interval, and each task. 95% are represented in the figure by the error bars attached to each mean point. AC, autocorrelation; ISI, inter-stimuli interval.

The RM ANOVA revealed only a significant main effect of the ISI, *F*_(4, 96)_ = 5.08, *p* < 0.001, $\eta_{p}^{2}$ = 0.17, with more negative mean windowed lag-1 ACs in the 1,000 ms ISI (*M* = −0.23, *SD* = 0.12) than in the 300, 450, 600, and 800 ms ISI (*M* = −0.15, *SD* = 0.13). Neither the main effect of the task nor the ISI × Task interaction were significant. Note that the effect size of the ISI main effect was larger than the required SESOI, which indicated that the effect was powerful enough to be considered as meaningful. Thus, the slower tempo induced more negative mean windowed lag-1 ACs (Figure 2), and this effect was similar across both tasks.

### 1.3. Discussion

The purpose of Study 1 was to examine the timing strategies used under different time and space constraints through the use of time series. The reported data showed that slow tempo favored negative ACs (i.e., predictive timing), regardless of the movement complexity. Notwithstanding, ACs were less negative when participants were constrained to move fast or close-to-SMT paces compared to when constrained to move slow. This pattern of results draws closer to an emergent timing strategy, which is characterized by an absence of corrective processes (Studenka and Zelaznik, 2008).

There was a general tendency to negative ACs in the present study. This observation is in line with previous studies using finger-tapping tasks (Pollok et al., 2005; Repp, 2005). It confirms the general need of cognitive processes for motor control, even in the simplest movements (Delevoye-Turrell et al., 2006). This phenomenon is probably due to the very nature of the tapping tasks, which requires the production of a series of discrete actions. Indeed, each finger movement has a distinct start and end, corresponding to the finger-screen contact duration. Hence, tapping may be considered as a cognitive task that requires the continuous updating of motor commands by feedback loops (Desmurget and Grafton, 2000; Guigon et al., 2008). However, these implementations take time; the findings that ACs were more negative at slow tempo suggest that in the absence of time constraints, predictive timing strategies can be implemented efficiently to further decrease both timing and spatial errors in task performances.

Overall, the results of Study 1 confirm that windowed lag-1 AC analysis is a mathematical approach that is powerful enough to reveal changes in the cognitive strategies for motor timing. It may be a proper tool to investigate the conditions triggering the alternation between emergent and predictive timing for adapted motor behavior. A key benefit of this method is that it takes into consideration the evolution over time of motor timing strategies (Delignieres and Torre, 2011), giving access to a sensitive analysis that avoids blurring of the data due to a single-averaging approach performed across an entire series. The data of Study 1 support the hypothesis that different timing strategies are implemented depending on the speed constraints of the performed movements. However, they do not inform on the cognitive nature of the timing process involved.

Past developmental research has suggested that executive functions are influential to modulate the speed of motor execution, and in particular to slow down the spontaneous pacing of voluntary motor actions (Provasi and Bobin-Bègue, 2003). A possible explanation is that the execution of slow movements requires further attentional resources to have an explicit representation and to memorize the time intervals to be produced. Thus, predictive timing would involve working memory to compare the memorized interval and the interval that is to be produced, at each step of the sequence (Treisman, 1963). In Study 2, the aim was to reveal the attentional cost associated to predictive motor timing. Our leading hypothesis was that acting slower than the SMT will require more cognitive resources than moving faster or close to one's natural SMT.

## 2. Study 2

Compared to other areas of cognitive neurosciences, it might appear that the mechanisms controlling our actions should be readily understood because the motor action outcome reveals its goal and gives access to its biological significance. However, adaptive movement involves much more than the contraction of a pre-defined sequence of muscles. Well-adjusted motor actions must be informed, not only by the constraints of the environment in which the movement is performed, but also by knowledge of the limits of our own biological system. This implementation is cognitively demanding even for simple actions performed a thousand times a day. The aim of Study 2 was to reveal the attentional demands of moving at various paces by means of a dual-task paradigm.

One of the most used experimental paradigms in psychology to reveal the attentional demand of an activity is the *dual task paradigm*, which consists in performing a task of interest concurrently with a secondary task. Because attentional resources are finite, the more expensive the primary task is in attention, the more the behavioral performance of the secondary task is impaired (Kahneman, 1973). The dual-task paradigm illustrates that attention is allocated on a moment-to-moment basis depending on task requirements. Through the years, experimental studies have demonstrated that the dual task paradigm is a valuable tool to reveal the dynamic nature of attention, managed by top-down control processes (Karatekin et al., 2004; Delevoye-Turrell et al., 2006).

In the following study, participants performed the same finger-tapping tasks as in Study 1, which was considered as the primary task. The visuomotor sequences were to be performed in synchrony with tempi ranging from 300 to 1,100 ms. In addition, a simple reaction-time task was included. To monitor levels of motor contraction, which reflects the degree-of-freedom and thus, the control strategy applied on the moving limb, finger pressure was also recorded.

### 2.1. Materials and Methods

#### 2.1.1. Participants

The sample size required for the present study was calculated using G*Power (3.1.9.2). The theoretical sample size was computed for a RM ANOVA. In the estimation of effect size, the dual task results of Brünken et al. (2004) were used as group parameters. The power analysis indicated that a minimum of 23 participants were required (*f* = 0.51; α = 0.05; 1-β = 0.80). An additional two participants were recruited in case of deletions due to outliers.

Twenty five right-handed participants between 18 and 35 years (*M* = 23.3, *SD* = 3.2) participated voluntarily in the study. Each of them received a participant information sheet, and completed a written informed consent. Participants reported having normal or corrected-to-normal vision and no deficiencies in terms of motor control.

As in Study 1, the small telescopes approach was used to determine the SESOI, and the dual task results of Brünken et al. (2004) were used as group parameters. The sensitivity analysis indicated that an effect size of at least *f* = 0.28 (i.e., $\eta_{p}^{2}$ = 0.07) was required to be meaningful.

#### 2.1.2. Tasks Description and Materials

##### 2.1.2.1. Primary Task

The two finger-tapping tasks were the same as in Study 1. The synchronization beeps indicated ISIs of either 300, 500, 700, 900, or 1,100 ms.

##### 2.1.2.2. Secondary Task

The secondary task employed in the present study consisted in the detection of a simple auditory stimulus (duration = 80 ms; sound frequency = 220 Hz). The participant was required to press a response button with her/his left hand as soon as the reaction time beep was heard. These beeps were presented to the participant six times within a trial, at random moments. Reaction time beeps could not appear during the first six, or the last three taps of a trial; two successive beeps were spaced by at least two taps. As in Study 1, auditive stimuli were generated using MATLAB 7.11.0 R2010 software (Mathworks Inc.; Natick, Massachusetts, MA).

#### 2.1.3. Procedure

Before starting the experimental session, three measurements of simple reaction time were performed. Then, a within-subjects design was applied, wherein each finger-tapping task was presented in a fully counterbalanced order.

For each of the two finger-tapping tasks, ISIs were randomly presented to the participant. A trial consisted in 60 synchronization beeps. Overall, participants performed a total of 10 trials. The total duration of the experimental test period was ~40 min. Participants were systematically debriefed at the end of the session.

#### 2.1.4. Data Acquisition and Processing

All collected data are available as Supplementary Material.

##### 2.1.4.1. Accuracy Indicators

To confirm that participants performed accurately the finger-tapping tasks, timing and spatial errors were computed. Before calculating accuracy indicators, the series of taps were checked to detect and remove data associated with IRIs greater than twice the ISI of a given trial. Overall, 0.5% of the data were rejected following this criterion. These trials were referred to as temporal omissions, and were not included in further analysis. As in Study 1, relative asynchrony (ms) and spatial error (pixels) were used as indicators of synchronization accuracy and spatial accuracy, respectively.

##### 2.1.4.2. Pressure

Pressure was estimated by the deviation matrix of the touchscreen (surface acoustic wave technology) that was coded between −32,768 and 32,768. Measured values were normalized on a scale going from 0 (no pressure) to 1 for the purpose of estimating the quantity of finger force applied on the touchscreen in the different experimental conditions.

##### 2.1.4.3. Secondary Task Performance

The shortest of the three reaction times (RTs) measured prior to the experimental test session was taken as the reference value for a participant. For the RTs performed under dual-task condition, ΔRTs were computed as the percentage of the reference reaction time. As an example, if the reference reaction time was 200 ms, and the dual-task reaction time was 250 ms, the ΔRT was 125%. A key benefit of this delta method is to suitably compare the reaction-time increase between participants, ignoring inter-individual variability. Before calculating the ΔRTs, RTs three times as long as the reference reaction time were removed from the statistical analysis. Overall, 3.4% of the data were rejected on this basis. Finally, the mean $\bar{\Delta }$RT was computed over a trial, and used as an indicator of the attentional demands required to perform the task.

#### 2.1.5. Statistical Analysis

To monitor the participants' performances in the two visuomotor tasks, the absolute asynchronies, the spatial errors and the pressure were submitted to a twoway, 2 (Task [1 target, 6 targets]) × 5 (ISI [300, 500, 700, 900, 1,100 ms]), RM ANOVA. To examine *H*_3_ and *H*_4_, the $\bar{\Delta }$RTs were also submitted to a twoway RM ANOVA. Normality (Shapiro–Wilk test) and sphericity (Mauchly's test) were checked. Tukey *post-hoc* tests were used as necessary. Statistica (v.13.1) was used for the statistical analyses, and the alpha level was set at *p* < 0.05.

### 2.2. Results

#### 2.2.1. Descriptive Results

Mean absolute asynchronies and spatial errors are presented in Table 2. To confirm that synchronization and spatial accuracies were consistent with the findings reported in Study 1, an additional ANOVA was run, with experiment (Study 1 vs. Study 2) as a between-subjects factor. The effect of experiment was non-significant for both the asynchronies, *F*_(1, 48)_ = 0.25, *p* = 0.622, $\eta_{p}^{2}$ = 0.054, and spatial errors, *F*_(1, 48)_ = 2.75, *p* = 0.104, $\eta_{p}^{2}$ = 0.005.

**Table 2:** Synchronization and spatial indicators for Study 2.

**Indicator**	**1-target pattern**					**6-target pattern**				
	**300 ms**	**500 ms**	**700 ms**	**900 ms**	**1,100 ms**	**300 ms**	**500 ms**	**700 ms**	**900 ms**	**1,100 ms**
Relative asynchrony	141	116	93	93	75	152	148	137	117	106
Spatial error	10.5	9.0	9.0	9.1	8.8	16.2	11.7	10.3	8.9	8.7

*Relative asynchrony (ms) and spatial error (pixel) for each inter-stimuli interval and each task*.

##### 2.2.1.1. Synchronization Accuracy

The RM ANOVA revealed a significant main effect of the ISI, *F*_(4, 96)_ = 9.97, *p* < 0.001, $\eta_{p}^{2}$ = 0.29, with the relative asynchrony decreasing steadily from the 300 ms ISI (*M* = 147, *SD* = 61) to the 1,100 ms ISI (*M* = 90, *SD* = 58). The main effect of the task was also significant, *F*_(1, 24)_ = 22.06, *p* < 0.001, $\eta_{p}^{2}$ = 0.48, with a smaller relative asynchrony in the 1-target (*M* = 104, *SD* = 67) than in the 6-target condition (*M* = 132, *SD* = 80). The ISI × Task interaction was not significant. Overall, these results indicated that participants made less timing errors as the tempo slowed down, and when the motor task was easy.

##### 2.2.1.2. Spatial Accuracy

The RM ANOVA revealed a significant main effect of the ISI, *F*_(4, 92)_ = 38.64, *p* < 0.001, $\eta_{p}^{2}$ = 0.63, with more spatial errors in the 300 ms ISI (*M* = 13.3, *SD* = 4.8) than in the 500, 700, 900, and 1,100 ms ISI conditions (*M* = 9.5, *SD* = 3.1). The main effect of the task was also significant, *F*_(1, 23)_ = 10.91, *p* < 0.001, $\eta_{p}^{2}$ = 0.32, with smaller spatial errors in the 1-target (*M* = 9.3, *SD* = 3.8) than in the 6-target condition (*M* = 11.2, *SD* = 3.1). The ISI × Task interaction was significant, *F*_(4, 92)_ = 12.30, *p* < 0.001, $\eta_{p}^{2}$ = 0.35. This interaction reflected that spatial errors were smaller in the 1-target than in the 6-target condition, but only at the 300 ms (*p* < 0.001) and 450 ms ISI conditions (*p* < 0.001); however, spatial errors were similar at the other ISI conditions. Overall, these results confirmed that the participants made more spatial errors when the motor task was complex, particularly when the speed of execution was faster than the SMT.

#### 2.2.2. Pressure

**Figure 3:**
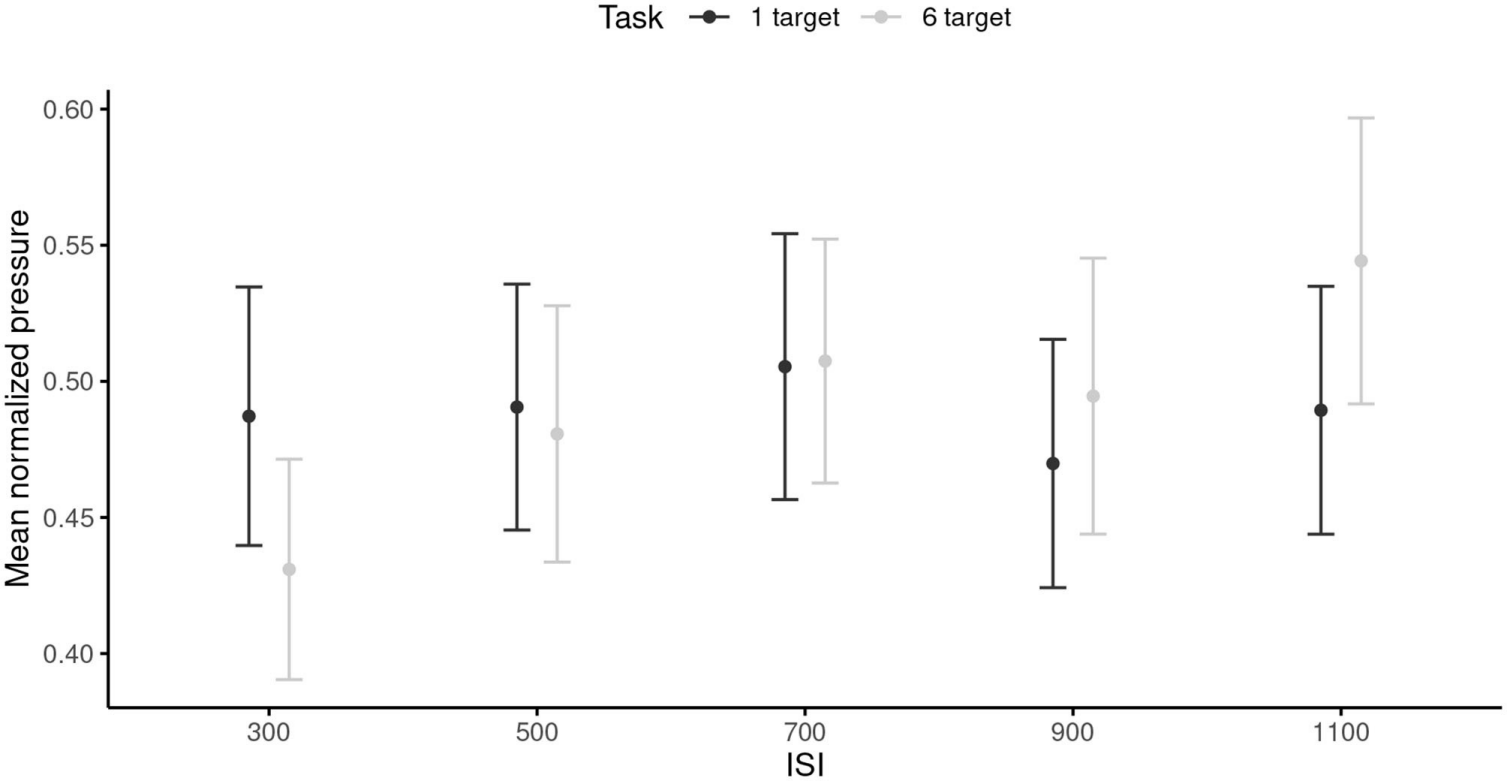
Mean pressure for each inter-stimuli interval, and each task. 95% are represented in the figure by the error bars attached to each mean point. ISI, inter-stimuli interval.

The RM ANOVA did not reveal any significant main effects of the ISI or the task. The ISI × Task interaction was significant, *F*_(4, 96)_ = 3.37, *p* = 0.019, $\eta_{p}^{2}$ = 0.12. This interaction reflected that pressure was smaller in the 6-target (*M* = 0.43, *SD* = 0.20) than in the 1-target condition (*M* = 0.49, *SD* = 0.24) in the 300 ms ISI condition (*p* = 0.014); however, the pressure was greater in the 6-target (*M* = 0.54, *SD* = 0.26) than in the 1-target condition (*M* = 0.49, *SD* = 0.23) in the 1,100 ms ISI condition (*p* = 0.017). Overall, these results indicated that the participants modulated the pressure applied to perform the task only in the 6-target condition (Figure 3).

#### 2.2.3. Secondary Task Performance

**Figure 4:**
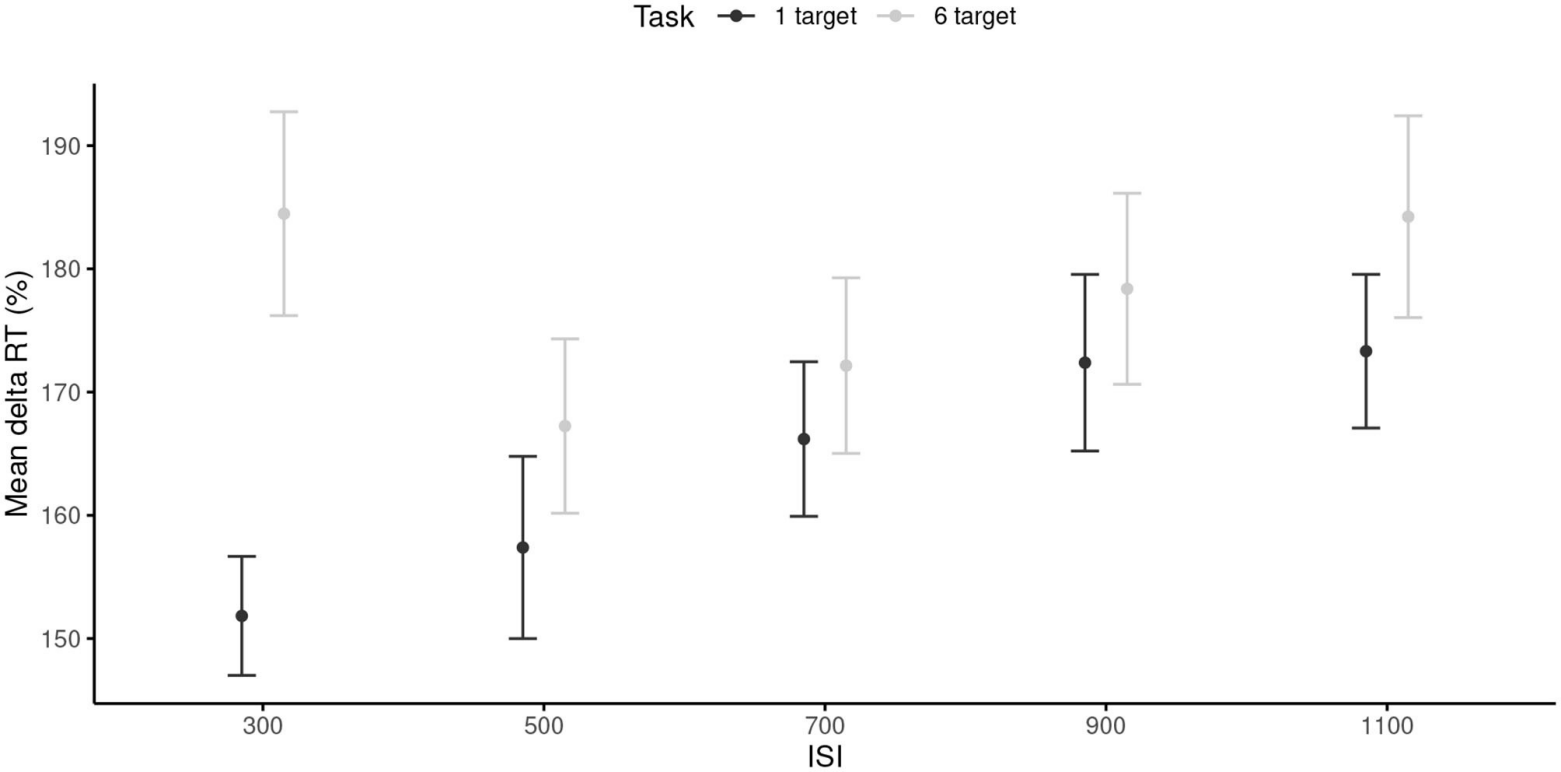
Mean 1RTs for each inter-stimuli interval, and each task. 95% are represented in the figure by the error bars attached to each mean point. RT, reaction time; ISI, inter-stimuli interval.

The $\bar{\Delta }$RTs were first tested against 100 with paired-sample *t* tests. To account for multiple comparisons, *p*-values were Bonferroni-corrected at *p* = 0.05/10 = 0.005. All comparisons were statistically significant (*p* < 0.001). The RM ANOVA conducted on the $\bar{\Delta }$RTs revealed a main effect of the ISI, *F*_(4, 96)_ = 7.33, *p* < 0.001, $\eta_{p}^{2}$ = 0.23, with shorter $\bar{\Delta }$RTs in the 300 and 500 ms ISI conditions (*M* = 165.14, *SD* = 34.45) than in the 900 and 1,100 ms ISI conditions (*M* = 177.07, *SD* = 36.67). The main effect of the task was also significant, *F*_(1, 24)_ = 44.38, *p* < 0.001, $\eta_{p}^{2}$ = 0.65, with smaller $\bar{\Delta }$RTs in the 1-target (*M* = 164.22, *SD* = 31.89) than in the 6-target condition (*M* = 177.30, *SD* = 38.40). The ISI × Task interaction was significant, *F*_(4, 96)_ = 9.27, *p* < 0.001, $\eta_{p}^{2}$ = 0.29. This interaction reflected the fact that $\bar{\Delta }$RTs were smaller in the 1-target compared to the 6-target condition, but only for the 300 ms (*p* < 0.001), 500 ms (*p* = 0.008), and 1,100 ms ISI tempi (*p* = 0.004; Figure 4). Note that the effect sizes of both the main effects and the interaction were larger than the required SESOI, which indicated that the effects were powerful enough to be considered as meaningful.

### 2.3. Discussion

The purpose of Study 2 was to examine the attentional demands of sequential movements performed at various paces by means of a dual-task paradigm. Both timing and spatial errors were similar to those presented in Study 1 and also to those previously reported in the literature of synchronization finger-tapping tasks (Dione and Delevoye-Turrell, 2015), indicating that participants were able here to perform the dual tasking without modulating the performance of the primary task. The findings of Study 2 demonstrated that motor production was always associated to a significant cognitive cost. The RTs were systematically greater in dual than single tasking ($\bar{\Delta }$RTs > 100). Tapping is a spontaneous movement that is sometimes observed in individuals who do not even realize that they are moving. The present findings are of interest for education because they indicate that suppressing this spontaneous body movement could help young individuals gain concentration by freeing cognitive resources (Nadeau and Rousseau, 1986).

Results of Study 2 revealed an effect of task complexity, with finger tapping in the complex conditions (6-target trials) engaging more attentional resources than finger tapping in the simple conditions (1-target trials). When the pointing was directed to a visual pattern of six targets, participants needed to control their pointing actions both through time and space. These requirements were in clear-cut contrast with that needed in the 1-target task, for which a simple one-joint movement of the finger was required, up-down in time with the external metronome. Figure 4 nicely illustrates that for the 1-target trials, the cognitive load increased proportionally with the decrease in tempo. For the 6-target trials, the attentional demands were important at both extremes, with a minimum around the SMT. This contrasting pattern is probably related to the fact that the 6-target trials required greater motor planning, preparation, and online control than the 1-target trials (see Paillard, 1985). Hence, motor-timing strategies were adapted to compensate for motor-task complexity. In the slower trials, attention may have been needed to maintain concentration on the task and manage slow body movements with both temporal and spatial constraints; in the faster trials, attention may have been used to simply manage to move the limb fast enough through space. The easiest pace was experienced at SMT for which both time and space were controllable with optimal sensorimotor loops.

Slowing down required cognitive resources. In both simple (1-target trials) and complex (6-target trials) finger-tapping tasks, the cognitive load of motor execution was more important when moving slow than when moving fast. The results of Study 1 indicated that finger tapping at slower tempi was characterized by more negative AC-1 than when tapping at faster tempi. We propose that this pattern of results suggest that slower voluntary actions are performed in the predictive timing mode, that is with the implication of greater cognitive control for each tap of the motor sequence. Findings from Study 2 confirm this interpretation by indicating that the slower the tempo, the more cognitive resources are needed. At the slowest tempo (i.e., 1,100 ms of ISI), cognitive control lead to an increase in muscle co-contraction that was quantifiable by a significant increase in the finger pressure on the screen. Overall, our results may reflect that attention is used to inhibit the urge to move spontaneously faster, hypothesis that is consistent with the proposed involvement of executive functions when decreasing the pace of voluntary movement (Provasi and Bobin-Bègue, 2003).

When moving at fast tempi, the amount of attentional resources needed to produce a voluntary movement varied depending on task complexity. In the 1-target trials, a simple one-joint movement of the finger was sufficient. Hence, correct performance was reached by creating a rigid body (wrist, finger, shoulder) with a single degree of freedom around the elbow. In such way, the central cognitive system did not need to regulate multiple articulations through space. Finger pressure remained constant across tempi, indicating that a similar biomechanical system was controlled with a focus on time. On the other hand, to perform the 6-target trials, participants needed to coordinate muscle contractions of upper limb through time and space. This required online corrective mechanisms in time intervals of different durations. In slower trials, individuals had time to apply the strategy to co-contract upper limb to gain a better control on the spatial accuracy of arm movement; this lead to an increase in finger pressure on the screen. However, in the faster trials, the burden of motor complexity on the attentional reservoir lead individuals to change timing strategies to prioritize space over time. This became visible in the fastest trials (300 ms of ISI), for which participants started to reach the biomechanical limits of the sensorimotor-control loops. Attention is absorbed in moving the hand through space to execute the finger-tapping task as best as possible. The loosening of limb articulations is provided to facilitate limb displacement, which led to a significant decrease in finger pressure on the screen.

## 3. General Discussion

For some years now, neuroimaging studies have argued for a differential control in emergent and predictive timing. The production of slow durations (i.e., sub-seconds intervals) recruit more strenuously prefrontal regions than fast durations (i.e., supra-seconds intervals), which preferentially involve motor and premotor areas (Lewis and Miall, 2003a,b). Yet, these *f* MRI studies failed to promote an explanatory hypothesis for the existence of a dual-timing strategy; actually, they merely reported differences in brain activation patterns according to interval duration in perceptual discrimination tasks. In the present contribution, a particular emphasis was set upon testing the nature of the cognitive process that are involved in the production of time intervals. Specifically, we adapted mathematical tools and the classic dual-task paradigm to shade light on the cognitive aspects of motor timing for tempi ranging from 300 to 1,100 ms of ISI.

### 3.1. A Dynamic Process

In the present research, the motor tasks were specifically designed to reflect contrasting complexity similar to that found in real-life situations. Everyone has already experienced eating popcorn while watching a movie. Moving the hand from the bucket to the mouth is easy and affords the peacefully enjoyment of the confectioneries while following the plot of the movie. Eating jelly pudding is another matter. Here, one must control the trajectory of the hand to lead the spoon to the mouth without spilling. Such movement will be performed more slowly and with much more attention; under such circumstances, it is hard to do something else at the same time. All attentional resources are focused on the smoothness of the movement. These examples nicely illustrate how the allocation of attentional resources is adapted in every-day activities to the constraints set by the environment on the motor system (see also Jones et al., 2002).

Using a unique behavioral task, it was possible to specifically examine the dynamic alternation between emergent and predictive timing under different timing and spatial constraints. Two key results surfaced from the data. First, the autocorrelations were negative. This observation can be explained by the fact that sequential pointing is a discrete task by nature. Nevertheless, the externally-imposed tempo constrained the way individuals controlled and performed the task. Indeed, at slower tempi, participants had more time to prepare each individual pointing movement. The times series were then even more negative; the cognitive cost of such online control increased. On the other hand, at faster tempi, less online control could be applied by lack of time. The time series were then less negative; the action was more automatized with fewer attentional resources allocated to the task. Hence, moving slow and fast yielded two contrasting patterns of behavioral results.

When an action is simple (e.g., pointing to a unique point in space), the same motor command can be triggered without adjustments, as similar muscle coordination can be simply repeated at a given pace. Individuals need only to manage the action temporally—trivially speaking, to decide when to trigger the release of the motor command. This way, regulation of movements is automatic (Maes et al., 2015), and does not require—or little—cognitive control, as motor actions are performed without the need of online feedback (i.e., open motor loop; Seidler et al., 2004). However, even in such simple cases, when slowing down motor tempo, the urge to move must be inhibited to be able to move at the right time as a function of task constraint. As a consequence, one needs to have a mental representation of the timing properties of the ongoing action (for a review of temporal-predictive processes, see Schwartze et al., 2012) and thus, to rely on internal loops to predict time of release as a function of sensory delays (Wolpert et al., 1995; Miall and Wolpert, 1996). Benefiting from an explicit representation of time, predictive timing allows for a cognitive monitoring of action performance. This could in particular imply the processing of the sensorimotor flow of information, working memory, and attention (Krampe et al., 2010; Berret and Jean, 2016). These processes are part of high-level cognitive control processes that are demanding in terms of resources. When such cognitive control is lacking, inadequate behaviors can be observed, as those found in impulsive disorders (see Wittmann et al., 2011; Grisetto et al., 2019). In the present study, participants possessed the capacity to inhibit the urge to move at a significant cognitive cost: under dual-task conditions, the reaction times were slowed down.

When an action is complex (e.g., pointing to different locations in space), individuals must deal with both time and space constraints. Pre-planning is not sufficient as the trajectory of each element will need to be adapted as a function of the errors performed on the previous element of the sequence. Hence, motor performances will take advantage of online corrections, which are implemented through sensorimotor reafferences that inform the central-cognitive system of performance errors. In the present work, the finger-tapping task in the 6-target trials needed to be spatially regulated. Hence, movement execution relied on the visuo-motor feedback loops to maintain performance accuracy (see Equation 1).

### 3.2. Critical Role of Available Attentional Resources

Generating a corrective signal for the motor commands is the outcome of the comparison between what is wanted (i.e., efference copy), and what was actually done (i.e., sensorimotor reafferences; Bard et al., 1992). Consequently, one needs to process the sensory information, compare them to that information which is stored in working memory, before updating the motor command and implement the required corrections. When moving slow, the system has the time to extract information from the control loops and implement correction on each and every element of the sequence, to minimize spatial and timing errors. When moving fast, the system does not have enough time to feed the motor loops in order to detect and correct errors—which is detrimental to effective movement realization. This is evidenced by the greater number of temporal and spatial errors. Because reaching the limits of the control loops, the motor peripheral system takes over at fastest tempi in the 6-target trials. The cognitive cost of motor control remains high, but is completed with a release of the body degrees of freedom (lower forces of impact) and a release of the predictive timing mechanism. Timing properties emerge from the motor patterns; an explicit representation of time is no longer used to guide motor timing, but the temporal properties emerge directly from the dynamics of the body in motion.

When the available attentional resources are sufficient to execute the motor plan and maintain the performance level, cognitive control of movement execution is feasible. However, when movement complexity and time pressure are too important, the temporal constraints and the degrees of freedom of motor control are adjusted to simplify motor execution and preserve timing accuracy. This motor-control simplification is further corroborate by the pressure force applied to perform the task. An increase in motor compliance—corresponding to a decrease in muscular tone—was measured when movement complexity and time pressure were high. It is possible that limb-control simplification entails a drop in the energy required to perform the movement. Indeed, individuals have a natural tendency to achieve the most cost-effective behavior (Selinger et al., 2015). The energetic *cost-minimization* framework states that energy cost continuously shapes movement (Cheval et al., 2018). Thus, individuals pursue a good balance between cognitive cost and efficiency. This fundamental principle is why there is sometimes a mismatch between “how well an organism can potentially perform, and how well that organism actually performs on a given task” (Inzlicht et al., 2018, p. 338). In the present work, we reported data from two studies that confirm the idea that timing properties emerge from body oscillations when the cognitive cost of motor control is too high due to movement complexity and/or time constraints. The biological system can then adopt the strategy to minimize the cognitive cost of moving by focusing on the *where* and letting body dynamics take charge of the *when*.

### 3.3. Limitations

There are two main limitations in the present contribution. First, different ISI conditions were used in Study 1 and 2. The decision to change the tempo conditions was taken because following Study 1, we felt that the time span between the fastest and the slowest conditions was not great enough—only the 1,000 ms ISI induced significantly more negative asynchronies when compared to the 300 ms ISI. Hence, a slower tempo condition (i.e., >1 s) was introduced in Study 2, while keeping the same number of ISI to limit fatigue. Second, the design of Study 2 did not include a single-task condition. This choice was made to constrain experimental session duration. This limited a truly meaningful between-experiment comparison. Future studies should target the inclusion of single-task control in dual-task paradigm when possible, and may use an ISI condition that matches each participant's spontaneous motor tempo to allow for inter-individual differences.

## 4. Conclusions

The findings of the present set of studies highlighted the significance of crossing mathematical and psychological tools to offer an holistic view of the cognitive processes involved in motor timing. Our data suggested that emergent and predictive timing may be underpinned by distinct mechanisms, that varies in cognitive control (see also Holm et al., 2017). Slow movements appeared to be more prone to cognitive monitoring (i.e., predictive timing), whereas fast movements seemed characterized by a release in control (i.e., emergent timing). Indeed, the introduction of a “cognitive control into the rigid machinery of sensorimotor habits” (Paillard, 1991, p. 248) takes time. The more time an individual has, the more a movement can be controlled, corrected, and set under the cognitive process of predictive-timing.

## Data Availability Statement

The datasets generated for this study can be found in online repositories. The names of the repository/repositories and accession number(s) can be found in the article/Supplementary Material. Datasets can be found here: https://osf.io/9vg53/?view_only=c88e8c0b327a438b933f56b89a7381fa.

## Ethics Statement

The studies involving human participants were reviewed and approved by the ethics committee of the University of Lille (France). The patients/participants provided their written informed consent to participate in this study.

## Author's Note

The study data and materials are shared openly as part of the publication of the article.

## Author Contributions

SG: conceptualization, methodology, investigation, statistical analysis, writing, project administration, and original draft. JB: software, formal analysis, visualization, review and editing. YD-T: conceptualization, formal analysis, writing, review and editing, supervision, and funding acquisition. All authors contributed to the article and approved the submitted version.

## Conflict of Interest

The authors declare that the research was conducted in the absence of any commercial or financial relationships that could be construed as a potential conflict of interest.

## Supplementary Material

Supplementary Figure 1 Graphical illustration of the effects of motor tempo on attention ressources required when executing simple or complex spatio-temporal motor tasks. Human individuals may experience contrasting psychological states as a function of the time constraints set upon motor execution.
