# Supplementary figures and images for: Attention Guides the Motor-Timing Strategies in Finger-Tapping Tasks When Moving Fast and Slow
Source: Front Psychol. 2021 Jan 25;11:574396. doi: 10.3389/fpsyg.2020.574396 (PMC7868383; doi:10.3389/fpsyg.2020.574396)

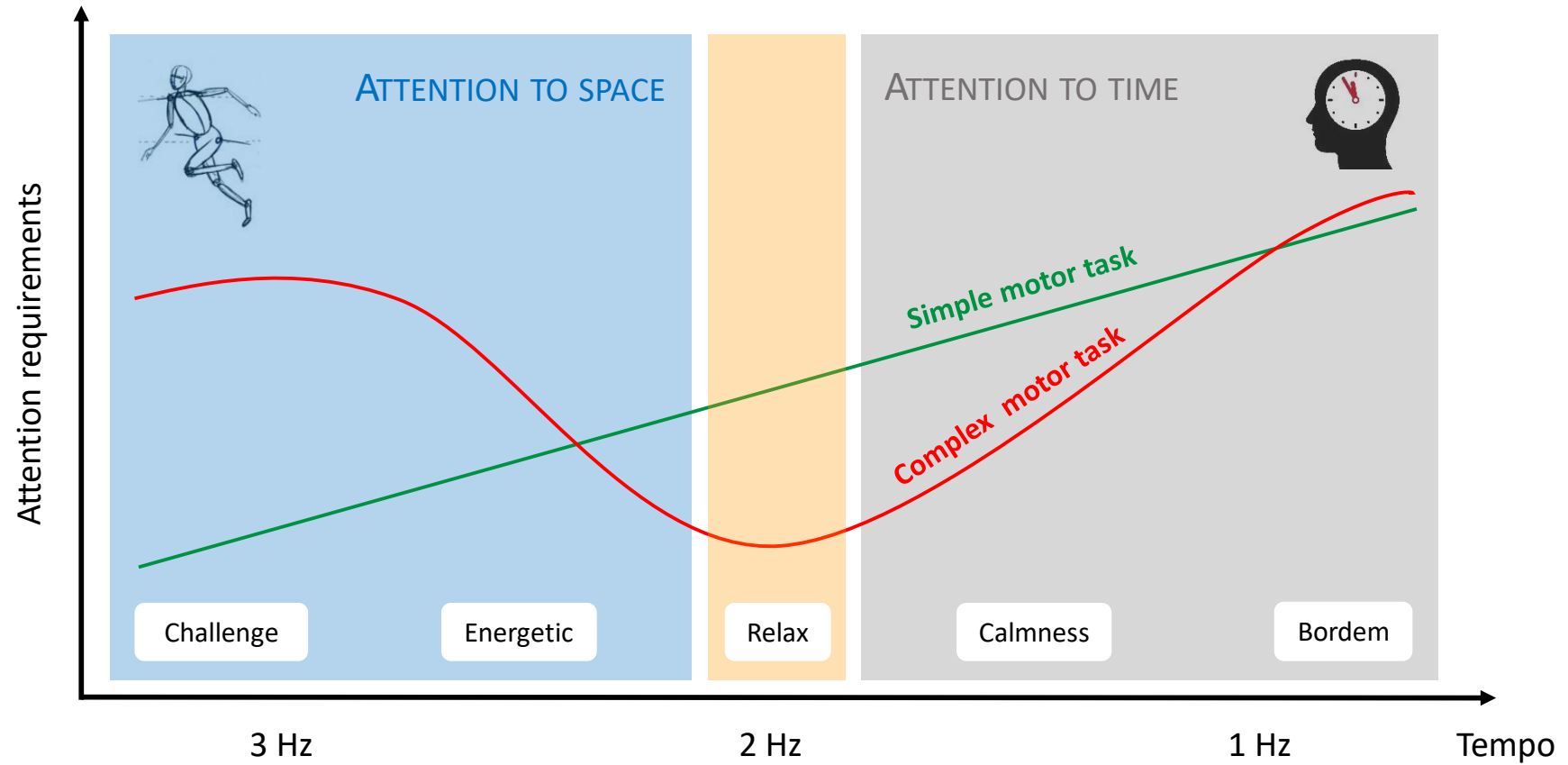

Supplement: Supplementary Figure 1 — Graphical illustration of the effects of motor tempo on attention ressources required when executing simple or complex spatio-temporal motor tasks. Human individuals may experience contrasting psychological states as a function of the time constraints set upon motor execution. [file Data_Sheet_1.PDF]
